# SNP discovery by high-throughput sequencing in soybean

**DOI:** 10.1186/1471-2164-11-469

**Published:** 2010-08-11

**Authors:** Xiaolei Wu, Chengwei Ren, Trupti Joshi, Tri Vuong, Dong Xu, Henry T Nguyen

**Affiliations:** 1 Division of Plant Sciences and National Center for Soybean Biotechnology, University of Missouri, Columbia, MO 65211, USA; 2 Beta Seed Inc., Tangent, OR 97389, USA; 3 Digital Biology Laboratory, Computer Science Department and Christopher S. Bond Life Sciences Center, University of Missouri, Columbia, MO, 65211, USA

## Abstract

**Background:**

With the advance of new massively parallel genotyping technologies, quantitative trait loci (QTL) fine mapping and map-based cloning become more achievable in identifying genes for important and complex traits. Development of high-density genetic markers in the QTL regions of specific mapping populations is essential for fine-mapping and map-based cloning of economically important genes. Single nucleotide polymorphisms (SNPs) are the most abundant form of genetic variation existing between any diverse genotypes that are usually used for QTL mapping studies. The massively parallel sequencing technologies (Roche GS/454, Illumina GA/Solexa, and ABI/SOLiD), have been widely applied to identify genome-wide sequence variations. However, it is still remains unclear whether sequence data at a low sequencing depth are enough to detect the variations existing in any QTL regions of interest in a crop genome, and how to prepare sequencing samples for a complex genome such as soybean. Therefore, with the aims of identifying SNP markers in a cost effective way for fine-mapping several QTL regions, and testing the validation rate of the putative SNPs predicted with Solexa short sequence reads at a low sequencing depth, we evaluated a pooled DNA fragment reduced representation library and SNP detection methods applied to short read sequences generated by Solexa high-throughput sequencing technology.

**Results:**

A total of 39,022 putative SNPs were identified by the Illumina/Solexa sequencing system using a reduced representation DNA library of two parental lines of a mapping population. The validation rates of these putative SNPs predicted with low and high stringency were 72% and 85%, respectively. One hundred sixty four SNP markers resulted from the validation of putative SNPs and have been selectively chosen to target a known QTL, thereby increasing the marker density of the targeted region to one marker per 42 K bp.

**Conclusions:**

We have demonstrated how to quickly identify large numbers of SNPs for fine mapping of QTL regions by applying massively parallel sequencing combined with genome complexity reduction techniques. This SNP discovery approach is more efficient for targeting multiple QTL regions in a same genetic population, which can be applied to other crops.

## Background

Identifying polymorphic markers associated with complex, economically important traits in crops has been hindered for most crops by the lack of whole genomic sequence, high-resolution maps and cost-effective platforms for high-density genotyping. In soybean, several thousands of genetic markers (mostly SSR and SNP markers) have been identified and mapped in the past ten years [1-4]. However, marker density is not enough to target candidate genes underlying a QTL region and conduct association mapping for complex traits. It was estimated that linkage disequilibrium extends for hundreds of kilobases in the cultivated soybean (*Glycine max*) group and that 10,000 - 75,000 SNPs would be necessary for whole genome associations in soybean [5]. Therefore, large numbers of markers and cost-effective SNP genotyping technology will make the whole-genome association analysis possible in soybean. A major limitation to the development of high throughput genotyping assays for soybean is the lack of SNPs covering the whole genome that can be used for genotyping. To date, approximately 5,000 public SNPs developed from soybean unigenes are available to the public [3]. The 8x shotgun sequence assembly of the soybean cultivar "Williams 82" has been released by the Department of Energy, Joint Genome Institute after the 6.5x scaffold assembly was initially released to the public http://www.phytozome.net/soybean. It provides an invaluable resource for soybean genome mapping and marker development [6]. Although we can design primers to target most of the sequences based on the available genome sequence, it is not feasible to develop hundreds of markers by sequencing PCR products using the Sanger method because it is costly, time consuming, and labor intensive [7].

With the recent advances in massively parallel sequencing technologies and the availability of whole genome sequence, the identification of a large number of polymorphic markers by re-sequencing different genotypes in model species such as human [8] and Arabidopsis [9], is changing the landscape of genetics, which can provide molecular genetic markers and insights into gene function [10]. The process of whole-genome re-sequencing using short-read technologies involves the alignment of millions of reads to a reference genome sequence and the determination of the variations in nucleotide sequence between the sample and the reference, based on statistics of multiple reads within an alignment. Currently, large-scale identification of SNPs by massively parallel sequencing technology for crop species is still a challenging endeavor because of genome complexity and the cost for whole genome deep-coverage re-sequencing.

For large and complex genomes, the identification of SNPs by massively parallel sequencing technology has been explored using reduced complexity sequencing approaches. There are several methods which aim to reduce the complexity of the sequencing template, in particular, by reducing the representation of low-information-content repetitive sequences. One such method is expressed sequence tag (EST) sequencing. For example, in the polyploid crop oilseed rape (*Brassica napus*), SNP discovery was exploited using Solexa transcriptome sequencing by aligning ESTs to the reference sequences, a set of publicly available unigenes [11]. However, as gene expression has temporal and spatial changes, the sample preparation is more challenging for SNP discovery because it requires the same set of ESTs generated from different genotypes that can be compared with each other. In addition, it is difficult to discriminate the orthologous sequences from paralogous sequences when working on a complex genome [12], due to gene duplication. As a result of these EST sequencing limitations mentioned above, the number of identified SNPs is relatively low in many species with validation rates usually between 50 and 85% [12].

SNP discovery for complex plant genomes by coupling the next-generation sequencing platform with a complexity reduction technique has been demonstrated in the CRoPS (complexity reduction of polymorphic sequences) system [13], which combines the 454 technology with the amplification fragment length polymorphism (AFLP) technique. Within the CRoPS system, tagged complexity-reduced fragment libraries generated by the AFLP process [14] was sequenced by 454 highly parallel sequencing technology, and the sequences were aligned and mined for polymorphisms. The system was validated with a 75% validation rate in maize [13]. SNP discovery by highly parallel sequencing was successfully demonstrated in cattle [15], in which reduced-representation pooled libraries were sequenced using the Illumina/Solexa technology. Alignment of the over five millions of 25-bp short reads to the bovine genome identified over 62,000 putative SNPs with a 91% validation rate. It is demonstrated that high-throughput sequencing of complexity reduced genomes for SNP discovery, with 8-20x sequence coverage, is a more efficient and effective way for the identification of large numbers of SNPs [15]. Taking advantage of the low cost of the approach, researchers are now applying this technique in other species to develop genome-wide SNP markers.

The feasibility of identifying SNP markers for QTL fine mapping by high-throughput sequencing at a low sequencing depth depends on the false call rate, genome coverage, and sequencing cost. To identify SNP markers in a cost effective way for fine-mapping of several QTL regions and determine the false call rate of putative SNPs at a low sequencing depth, we evaluated a pooled DNA fragment reduced representation library and the SNP detection method applied to short read sequences generated by Solexa massively parallel sequencing technology.

## Results

### Test restriction digestions

**Figure 1:**
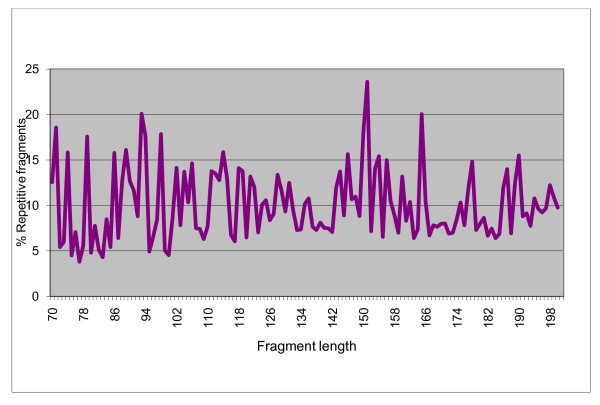
**The proportion of fragments containing repetitive elements in the targeted fragments**.

**Figure 2:**
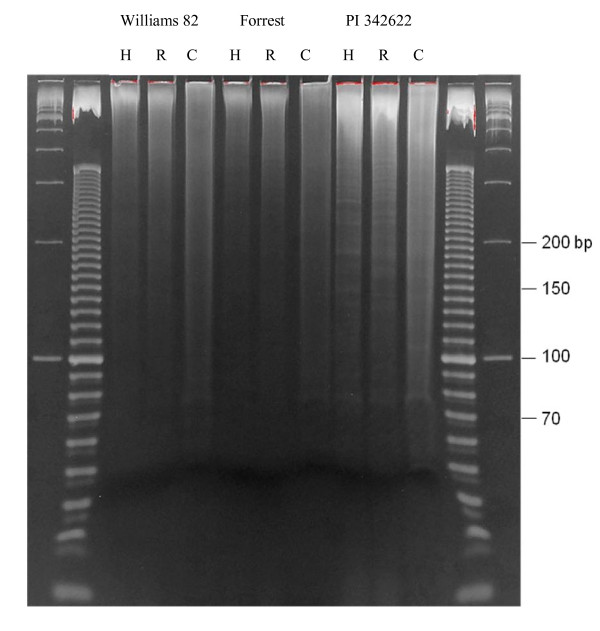
**DNA digested by different enzymes**. H: *Hae *III; R: *Rsa *I; C: *CviR *I

Based on *In silico *analysis results (Table 1), the *CviR *I restriction enzyme showed to be the best one for construction of the reduced representation genomic DNA library due to its more uniform size distribution of digested fragments, relative high number of fragments in the size range (70-200 bp) as we expected [15], and no major repetitive element peaks in the diagram showing the repetitive fragment percentage over the selected size range (Figure 1). To verify *in silico *digestion results we tested the real digestions of genomic DNA of Forrest, Williams 82, and PI 342622 (*G. soja*) with three enzymes (*CviR *I, *Hae *III, and *Rsa *I) which produced the number of fragments more close to what we expected (Figure 2). It was shown that the restriction digestion for different genotypes by *CviR *I was much better than that by *Hae *III and *Rsa *I.

**Table 1:** Summary of DNA *in silico *digestion of soybean genome assembly

Enzyme	# total fragments	# selected fragments (70-200 bp)	total length	complexity reduction %	coverage for one run	sequence density per Mbp	putative SNP #	% selected portion	# repetitive in selected fragments	% selected fragments containing repetitive elements
*Acc *II	454,161	72,612	9,221,724	0.84	208.66	132.02	958.48	15.98	2332	0.51
*Hae *III	1,342,945	265,289	33,691,703	3.06	57.11	482.34	3,501.81	19.75	12,089	0.90
*Rsa *I	1,731,973	334,265	42,451,655	3.86	45.33	607.75	4,412.30	19.29	26,105	1.51
*Dpn *I	2,475,326	594,055	75,444,985	6.86	25.51	1,080.10	7,841.53	23.99	62,906	2.54
*Alu *I	2,929,186	841,548	106,876,596	9.72	18.00	1,530.09	11,108.43	28.72	66,175	2.26
*CviR *I	4,267,589	1,367,611	173,686,597	15.79	11.08	2,486.57	18,052.47	32.04	140,580	3.29
*CviJ *I	8,002,903	2,470,067	313,698,509	28.52	6.13	4,491.03	32,604.88	30.86	189,866	2.37

### Library construction and quality control

The equally pooled DNA sample of Forrest and Williams 82 was digested with *CviR *I and the fragments in the 70-200 bp size range were isolated from a polyacrylamide gel (Figure 2). The fragment library was constructed and 1.8 μg final products were obtained. To verify the quality of the library, a portion of the final DNA products were cloned into a plasmid vector and randomly selected 192 clones for one direction sequencing on the ABI 3730 sequencer (Applied BioSystems, Foster City, CA). Then, these sequences were aligned to the soybean genome assembly. Of the 154 good quality sequences, 74 (48.1%) sequences were locus-specific, 27 (17.5%) sequences had multiple hits in the genome, and 53 (34.4%) sequences had no hit on the 6.5x scaffold assembly.

### DNA sequencing and SNP discovery

**Figure 3:**
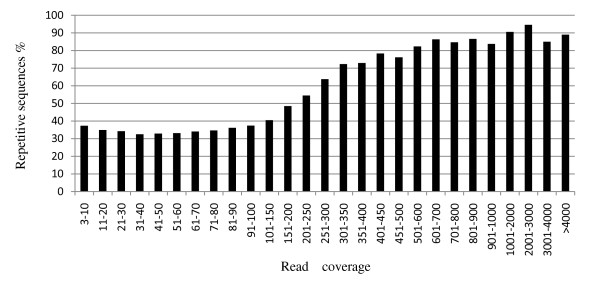
**repetitive sequence proportion in the sequences with different read coverage**.

The pooled library was sequenced in two flow cells, totally generating over 42 million reads (1.47 Gb) corresponding to 9.46 million unique 33-mer sequences, resulting in an average coverage of 4.4x. However, the sequence clusters were seriously biased. It was shown that 72.7% of the 33-mer sequences were just single read, 24.4% of those had multiple reads, and 2.9% of unique sequences were low quality sequences containing at least one unknown nucleotide 'N'. After removing 295,788 low-quality sequences, over 41.7 million 33-mer reads passed the standard Illumina filtering and more than 40 million reads (91.6% of high quality reads) could be aligned to the reference Williams 82 genome sequence, 6.5x scaffold assembly. Finally, 6.8 million sequences had a single read and 2.3 million sequences with multiple reads remained for SNP discovery. When plotting the depth-coverage of those sequences used for SNP discovery over the chromosome coordinates, the distribution exhibited relatively uniform across the whole genome (data not shown). However, we noticed that the distribution of read coverage was extremely biased (Additional File 1), which revealed overdispersed Gamma distribution [16]. In the sequences with multiple reads, 60% of them were sequences with 10 reads or less. The sequences with more than 200 reads was a small portion (0.6%) but their sequence reads accounted for 27.6% of the total reads of sequences with more than 3 reads. By analyzing sequence characteristics (repetitiveness and GC content) and number of reads in a sliding window of 10 kbp in width, we found a correlation of read coverage and percentage of repetitive sequences in the 2.3 million sequences with multiple reads. The result showed that 70.2% of the sequences with more than 200 reads were located in repetitive regions, but only 36% of sequences with less than 200 reads were repetitive (Figure 3). There was no correlation between depth-coverage and GC-bias (R = 0.029).

**Figure 4:**
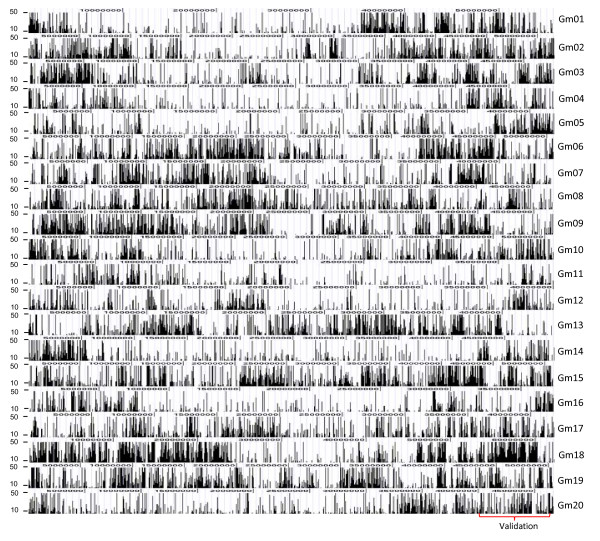
**Distribution of putative SNPs on the 20 chromosomes of the reference genome**. The *x*-axis represents SNP read coverage, and the y-axis represents chromosome coordinates of the reference genome.

To clearly evaluate the feasibility of SNP discovery using Solexa high-throughput sequencing, two different levels of stringency (see details in Materials and Methods) were initially used for SNP prediction and filtering. The less stringent parameters identified 39,022 putative SNPs between the Solexa short-reads and the 6.5x assembly with an average SNP density of 31.5 Kbp across the entire genome, which were not uniformly distributed across soybean chromosomes, especially more SNPs were clustered in gene-rich, high-recombination euchromatic regions than in repeat-rich, low-recombination heterochromatic regions (Figure 4). The number of the predicted SNPs was not evenly distributed over the 33-mer positions (Additional file 2). The highly stringent filtering method predicted 10,874 putative SNPs on the 6.5x scaffold assembly, which were further analyzed against the newly released Williams 82 soybean genome sequence, 8x scaffold assembly. We aligned the original short-read sequences containing the putative SNPs onto the 8x scaffold assembly using BLASTN and excluded SNPs which had more than one position in the genome. We also identified the repeat sequences in the 8x scaffold assembly using RepeatMasker http://repeatmasker.org and aligned the original short-read sequences containing the putative SNPs onto the repeat masked scaffold assembly. SNPs which short-read sequences could not be aligned to the repeat-masked genome sequence were also excluded. It indicated that these SNPs might be in the repetitive regions in the genome or they failed to align to the 8x scaffold assembly due to some sequence changes from the 6.5x scaffold assembly.

**Figure 5:**
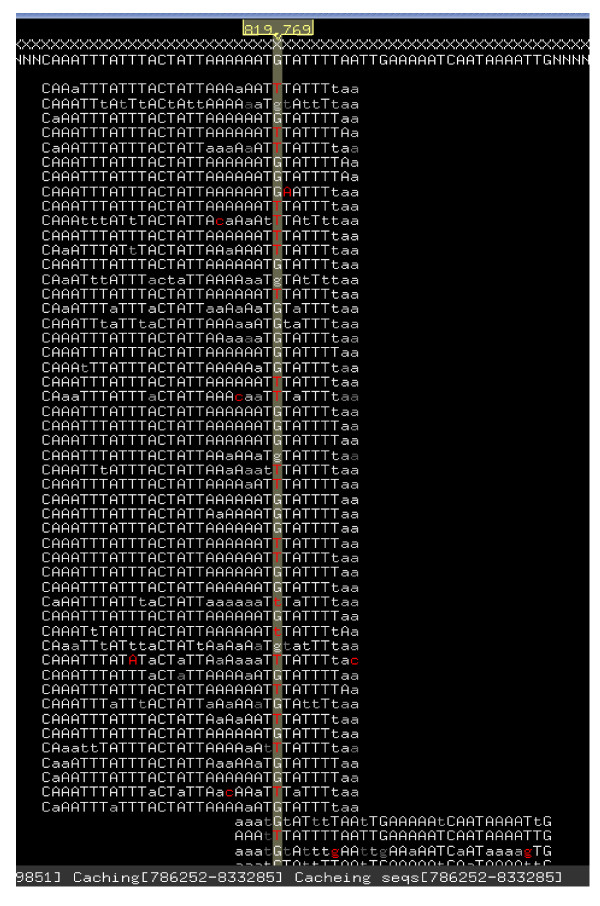
**Visualization example of alignments of short read sequences to the soybean reference genome**.

Finally, 7,947 SNPs were identified after filtering with the highly stringent criteria as well as aligning onto the 8x scaffold assembly (Additional File 3). Some of the SNPs were also examined by loading the alignments of short-reads into Maqview http://maq.sourceforge.net/maqview.shtml for visualization (Figure 5). The flanking 60 bp sequences around the SNP position were extracted for the SNP array design.

### SNP validation

To evaluate stringency parameter sets for SNP prediction and the feasibility of this SNP discovery approach for fine mapping, , we deliberately selected predicted SNPs for validation from the list of 39,022 putative SNPs predicted on the 6.5x scaffold assembly, and then applied more strict parameters for SNP calling to see how the stringency setting would affect the validation rate. We selected an 8 cM QTL region associated with soybean cyst nematode resistance in chromosome 20 [17]. This QTL was in a repeat-poor, gene-rich euchromatic genomic region, which had only 8.9% repetitive sequence, while the soybean genome is made of ~59% repetitive elements [6]. A total of 354 putative SNPs (average 25.8 Kbp apart) were predicted in the 8 cM QTL region, we selected 250 SNPs with at least 10 kb intervals for validation, The flanking sequences derived from the 6.5x scaffold sequence were used for primer design, generating 400-500 bp amplicons for resequencing by Sanger method. Out of 250 PCR amplicons, eight primer sets failed to amplify clear PCR product. Ten primer sets amplified multiple products that caused the sequencing reads to be noisy. Thus, 232 primer sets generated good quality of sequences both for Forrest and Williams 82. By aligning the sequences of the two genotypes, of the 232 sequence tagged sites (STS) containing the predicted SNPs, 164 STSs containing the putative SNPs were validated, which resulted in a 72% validation rate.

Validation rate is important for high throughput SNP genotyping. To increase the validation rate, we set up more stringent parameters for SNP calling as mentioned above. Out of the 232 primer pairs that produced a good quality amplicon, only 61 were remained in the final list of 7,947 SNPs predicted with higher stringency. According to the validation data, 52 of the 61 produced amplicons containing the predicted SNP, which led to an 85% validation rate. We noticed that 171 of the 232 primer pairs initially selected were filtered out mostly due to the number of sequence reads low than 10 and potential multiple locations in the genome, but still having a 66.1% (113/171) validation rate. The validation rates in different scenarios provided a useful guideline for SNP predictions and selection of SNPs for different applications.

## Discussion

Fine-mapping multiple targeted QTL regions requires a large number of molecular markers that could be used to narrow down the regions and pinpoint candidate genes or target markers associated more closely with traits. Our objective was to design a solid experiment to test whether we could develop 50-200 markers within any targeted QTL region (5-20 cM) using high-throughput sequencing technology. Our results indicated that massively parallel sequencing technologies could offer an opportunity for simultaneously developing large numbers of high quality SNPs within target mapping populations. These SNPs could serve to fine-map the QTLs that were identified in QTL mapping studies or to conduct association mapping. Especially, with the improvements in sequencing output, paired-end reads and multiplexing techniques, the massively parallel sequencing technologies makes the whole genome SNP discovery more effective and efficient.

In theory, any genome complexity reduction strategies could be applied to generate less complex genomic DNA libraries for high-throughput sequencing. Especially for complex genomes such as maize, wheat, barley and soybean, genome complexity reduction is an important step so that genome-wide polymorphism discovery would become successful in organisms with high levels of repetitive DNA in the genome and/or low levels of polymorphism in breeding germplasm. Compared to methylation complexity reduction approach, restriction enzyme-based methods may be more appropriate for SNP discovery, because most of the short fragments generated by restriction enzymes from different genotypes would have a perfect alignment. The complexity reduction approach can be optimized by identifying an appropriate restriction enzymes using *in silico *digests of a draft genome sequence and test digest. Our results indicated that *CviR *I was the best enzyme for soybean. Of course, selection of one or multiple restriction enzymes for reduced representation library also relies on genome features in a given organism and applications. For example, *Hae *III was used for construction of bovine reduced representation libraries for deep sequencing by the Solexa platform [15]. Recently, five restriction enzymes were used for construction of a reduced representation library in a wild soybean (*Glycine soja*) for SNP discovery to help soybean sequence assembling [18].

The pooling of DNA samples from the parental lines for the target population, combined with the high-throughput sequencing of a small fraction of the genome results in the identification of a large number of SNPs genome wide. Most importantly, this approach also allows us to develop a large number of SNP markers for less well characterized species in an efficient and cost-effective way. Especially, with the increase of the output of highly parallel sequences and the decrease of the cost per run, it is more feasible to get deep sequences for multiple individual genotypes or pooled samples. However, due to a few drawbacks of pooling the DNA samples, the pooling strategy may not be necessary for sample preparation for duplicated genomes. For duplicate genomes such as soybean and maize, a portion of putative SNPs are derived from the alignment of paralogous sequences, i.e. the sequences aligned may come from different loci that are duplicated through evolution in the genome. The 85% SNP validation rate in our pooled sample, even at a high stringency level, indicated that 'paralogous' SNP is a problem for pooled samples. But it can be overcome by using a barcoding technique. It is not a problem to sequence many samples in a Solexa instrument run if using a barcoding technique. It is becoming a standard approach to increase the number of samples run on high-throughput instruments [19].

Comparing to the current Solexa sequencing system, our sequencing data were limited in terms of sequence length, accuracy (paired-end) and output because the super-high throughput Solexa sequencing system was not available when we conducted this experiment. Therefore, the depth-coverage of the short sequence reads was not enough to take account of allele frequency in SNP calling [15]. Paired-end reads definitely increase the power to detect sequence variations and improve the placement of most short reads to the genome which would dramatically reduce errors of SNP prediction caused by sequencing and misplacement of short reads [20].

In addition, the short reads cannot be assembled to give complete sequence of complex plant genomes [21] and repeat sequences cannot be aligned unambiguously with the reference genome [22]. For example, approximately 8.4% of the sequence reads cannot be aligned unambiguously with the Williams 82 genome, despite that the Williams 82 genome sequence, 8x Scaffold assembly, had 97% of genome sequenced. This portion of sequences may be unique in Forrest but not present in Williams 82, or in the gaps of the assembly. If some of them came from exons, they must be unique genes that may serve as causative variations for some traits. Some novel technologies that produce longer and paired-end sequences can improve SNP calling and facilitate the identification of indel markers by massively parallel sequencing technologies [23]. In addition, it was demonstrated that using quality score and longer reads can improve accuracy of Solexa sequence mapping to the reference genome [24].

We noticed that the threshold settings were important for SNP prediction. The more stringent thresholds gave a higher validation rate. But we had to balance the number of predicted SNPs that could be used for fine mapping and the validation rates. We may also take account of the SNP distribution along the 33-mer Solexa sequence positions (see Figure 4), especially SNPs occurring in the first few nucleotides of short-reads might be overrepresented with a high percentage of predicted SNPs. However, further analyses are needed to validate these SNPs over different positions in the 33-mer sequences for fine-turning the filtering parameters used for SNP prediction.

To explain potential causes of the observed sequence biases, a comprehensive analysis of the Solexa sequencing procedures and effects of sequence characteristics is necessary. We did observe a strong correlation between percentage of repetitive sequences and read coverage, with the read density being increased in repetitive regions. However, we did not observe a correlation between GC content and read coverage, which is known to be present in Solexa sequencing DNA samples prepared by nebulization or sonication [25]. Such biases have implications on the utilization and interpretation of Solexa sequencing data for the identification of SNPs and transcriptome profile analysis.

## Conclusions

Massively parallel sequencing technologies enabled us to generate a large amount of sequences in the complex genome - soybean, which allowed us to identify many SNPs in QTL regions of interest. We have demonstrated an efficient and economical way to develop SNP markers for QTL fine-mapping and provided a guideline for methods used in preparing complexity-reduced genomic DNA sequencing samples and selecting parameters for sequence data filtering and SNP prediction. We have applied this approach to fine-mapping multiple QTLs related to soybean cyst nematode resistance, and constructing a high resolution of genetic map.

## Methods

### *In silico *restriction digestion and enzyme selection

*In silico *digestion of the soybean genome sequence assembly, 6.5x scaffold assemby http://www.phytozome.net/soybean were performed with seven commercially available 4-cutter DNA restriction enzymes generating blunt end fragments. The enzymes were evaluated from the following parameters: 1) total number of fragments predicted, 2) number of fragments predicted in the target size range (70-200 bp), 3) proportion of repetitive nucleotides in the terminal 33 base pairs of each fragment end, and 4) number of putative SNPs predicted. The identification of repetitive elements was based on the soybean repetitive sequence database and annotation of soybean genome browser http://www.phytozome.net/cgi-bin/gbrowse/soybean/. The number of putative SNPs was predicted based on the SNP frequency in the soybean genome [3].

### Library construction

Genomic DNAs of cultivars "Forrest" and "Williams 82" were used for the reduced representation genomic DNA library construction. The genomic DNA concentration of each sample was measured by the Pico green assay with the Quant-iT ds DNA Assay Kit (Invitrogen, Cat. No Q33120) per manufacturer's instructions. Equal amounts of the two genomic DNAs were pooled and 6 μg pooled DNA was digested by *CviR*I (New England Biolabs) with 60 units of enzyme in a total volume of 150 μl, as suggested by the manufacturer. The digestion proceeded overnight at 37°C to ensure complete digestion. The resultant fragments were size fractionated on a 6% non-denaturing polyacrylamide gel (16 cm × 14 cm, 1.5 mm thick), stained with Syber Gold (Invitrogen, Cat. No S11494), and imaged under a Dark Reader. The digested products between 70 and 200 bp were excised and sliced into smaller pieces. The gel pieces were sheared by squeezed through a hole punctured by an 18-gauge needle at the bottom of a 0.5 ml microcentrifuge tube, with the help of centrifuging at 16,000 g for 2-3 min. The sheared gel was collected into a 2 ml microcentrifuge tube, in which the above 0.5 ml tube was nested. The DNA fragments in the sheared gel were recovered by elution into 2x of volume of DNA elution buffer (8 mM Tris pH 8.0, 0.08 mM EDTA, 1.25 M ammonium acetate) at room temperature overnight, followed by a 15 min incubation at 65°C. The eluted DNA fragments were transferred into a 15 ml falcon tube after centrifuging at 16000 g for 2 min and a couple of times washing with the elution buffer. The eluted DNA was then purified with a SNAP column (Invitrogen, Cat. No 45-0078), and precipitated by 2.5x volume of 70% ethanol at -20°C for 1 h, followed by centrifuging at 4000 g for 15 min. The pellet was twice washed with 500 μl cold 70% ethanol, dried, and resuspended in 50 μl of TE (10 mM Tris pH 8.0, 0.1 mM EDTA). DNA quality and quantity were checked by reading O.D. on a Nanodrop spectrophotometer.

The blunt-ended DNA fragments produced by *CviRI *digestion were treated with Klenow fragment of DNA polymerase lacking exonuclease activity in the presence of dATP to add an adenine overhang to the 3' ends of each strand. This reaction was performed in a 50 μl reaction volume using the buffer and the enzyme in the kit provided by the supplier (Illumina, Inc., Cat. No 1000181) at 37°C for 20 min, followed by inactivation of the enzyme at 70°C for 5 min. The modified fragments were precipitated with ethanol in the presence of 0.5 μg/μl final concentration of glycogen carrier, dried, and resuspended in 10 μl TE. Adapters required for sequencing on the Illumina CSMA/SBS platform were added to each DNA fragment by ligation in a 50 μl volume, using adapters and buffers supplied in the kit (Illumina, Inc., Cat. No 1000181). Ligation proceeded for 15 min at 22°C to produce fragments with 65 bases added from the adapter primers.

The ligation products were separated on a 6% non-denaturing polyacrylamide gel as described above. The ligation products in the size range from 130 to 190 bp were excised, eluted from the gel slice, precipitated and resuspended in 15 μl TE, using the same methods described above. The adapter-modified DNA fragments were amplified as per the instructions of the manufacturer's kit. The amplified products were purified using a Qiaquick PCR Purification Kit (Qiagen, # 28108) and collected in 50 μl of the provided buffer. After quantification by O.D. reading, calculation of molar concentration and quality examination (as described below), this PCR product of the reduced representation genomic DNA library of pooled Forrest and Williams 82 was shipped to Illumina for sequencing.

### Library quality control

Before submitting to Illumina for sequencing, the quality of the library was determined by cloning a portion of DNA fragments into the pPCR-Script Amp SK(+) Cloning Vector provided in the PCR-Script Amp Cloning Kit (StrataGene, #211188), per manufacturer's instruction, with the molar ratio of insert to vector DNA ranges at 50:1. The insert-vector DNA was transformed into the Maximum Efficiency DH 10 B Competent Cells (Invitrogen, #18297010) according to the manufacturer's instruction. The plasmid DNAs were extracted by alkaline lysis method, and then sequencing reaction was prepared with BigDye^® ^Terminator v3.1 Cycle Sequencing Kit (Applied Biosystems, Foster City, CA), followed by sequence analysis on the ABI 3730. Sequence traces were analyzed and aligned with software CromasPro v1.5 http://www.technelysium.com.au/ChromasPro.html.

### Solexa sequencing

The library constructed from Forrest and Williams 82 was loaded onto two independent flow cells. Quality scores were generated from Illumina's data analysis pipeline and are similar to SAGE Phred scores and have a maximum value of 40. Quality scores are based on the relative confidence of base calls using elements of cluster generation and image quality.

### Genome alignments and SNP detection from Solexa sequencing data

The Forrest and Williams 82 pooled Solexa sequencing data from two flow cells were combined. The Williams 82 6.5x scaffold assemblies were acquired from Phytozome http://www.phytozome.net/soybean. SNP prediction was initially performed using MAQ mapping and assembly software [26]. To determine filtering parameters used in SNP prediction that could produce suitable number of SNPs with an acceptable validation rate for fine mapping, we compared two different levels of stringency in the SNP prediction. The less stringent criteria were used in the filtering for SNP prediction: 1) SNPs with less than three short-reads were eliminated, 2) SNPs located in repetitive regions of the reference genome were eliminated, 3) filtered out SNPs if three or more SNPs appear in any 10 bp window, and 4) discarded any short-read sequences with an "N". The more stringent criteria were applied for SNP prediction: 1) SNPs within 3-bp flanking region around a potential indel were discarded, 2) SNPs had to be supported by at least ten short sequence reads, 3) discarded SNPs covered by no read with a mapping quality score higher than 40 in any 10 bp window, if there are 3 or more SNPs, discarded them all, 4) SNPs with consensus quality smaller than 10 were eliminated. The SNP data have been deposited in Soybean Knowledge Base http://soykb.org/.

## Authors' contributions

XW designed and coordinated the study, performed primer design and data analysis, and drafted the manuscript. CR created the reduced representation library, and assisted the manuscript. TJ and DX performed SNP prediction analysis, and assisted the manuscript. TV performed SNP validation resequencing. HTN designed and oversaw the study, and assisted in preparing the manuscript. All authors read and approved the final manuscript.

## Supplementary Material

Additional file 1 **Histogram of read coverage for the sequences with 3 reads and more**. This figure revealed the distribution of read coverage in bins (bin width = 1). The *x*-axis displays different value of read coverage and the y-axis displays the possibility of a particular read coverage. Click here for file

Additional file 2 **Distribution of putative SNPs over the different positions of the 33-mer short-read**. The *x*-axis represents the number of the predicted SNPs, and the y-axis represents the position of the 33 short-read. Click here for file

Additional file 3 **Distribution of 7947 SNPs**. This figure showed the distribution of the 7,947 SNPs predicted by strict stringency on the reference genome. The *x*-axis represents short read coverage, and the y-axis represents the SNP position on the chromosomes of the reference genome. Click here for file
