# Supplementary figures and images for: SNP discovery by high-throughput sequencing in soybean
Source: BMC Genomics. 2010 Aug 11;11:469. doi: 10.1186/1471-2164-11-469 (PMC3091665; doi:10.1186/1471-2164-11-469)

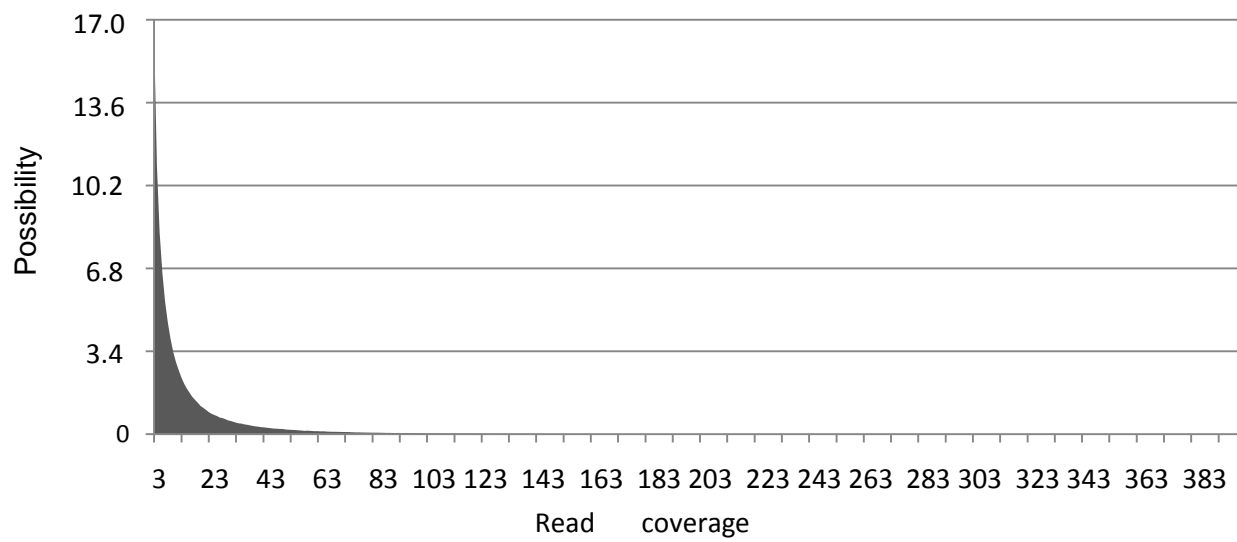

Supplement: Additional file 1 — Histogram of read coverage for the sequences with 3 reads and more. This figure revealed the distribution of read coverage in bins (bin width = 1). The x-axis displays different value of read coverage and the y-axis displays the possibility of a particular read coverage. [file 1471-2164-11-469-S1.PDF]

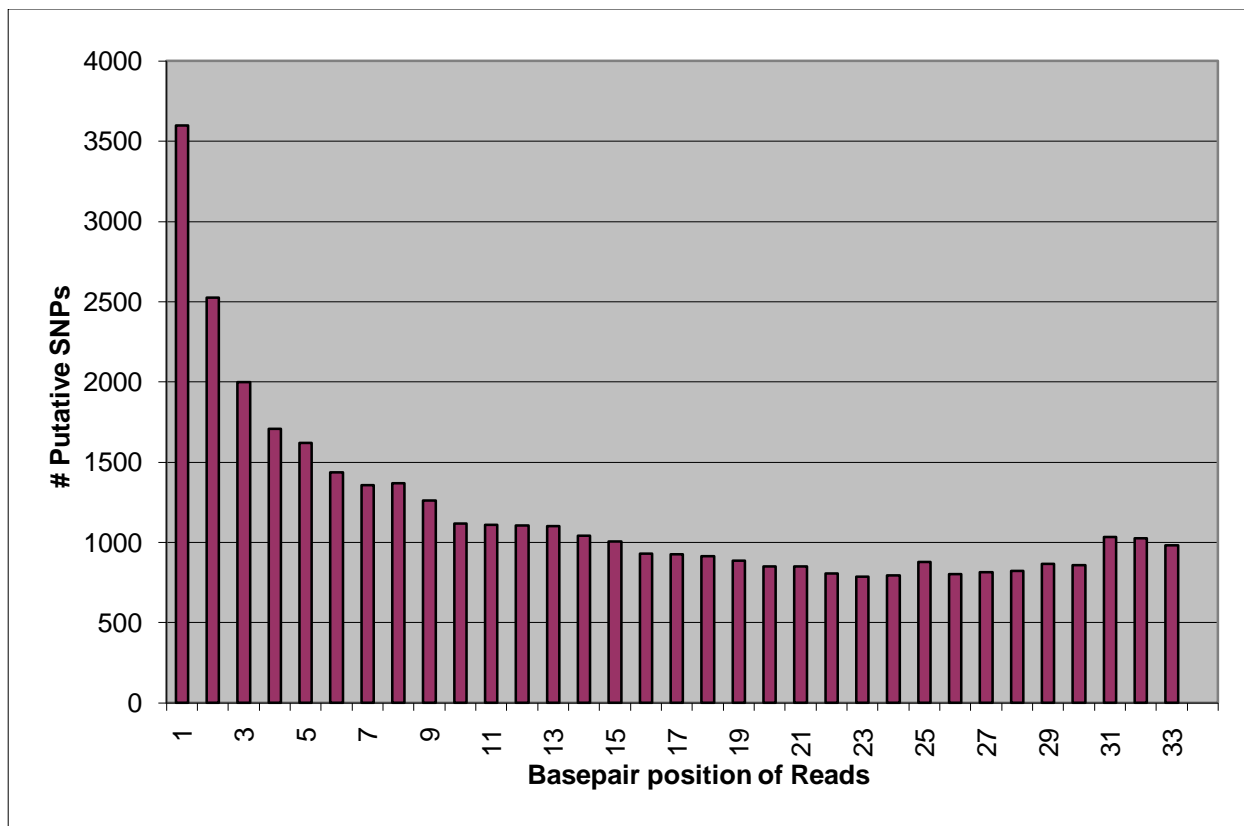

Fig3

Supplement: Additional file 2 — Distribution of putative SNPs over the different positions of the 33-mer short-read. The x-axis represents the number of the predicted SNPs, and the y-axis represents the position of the 33 short-read. [file 1471-2164-11-469-S2.PDF]

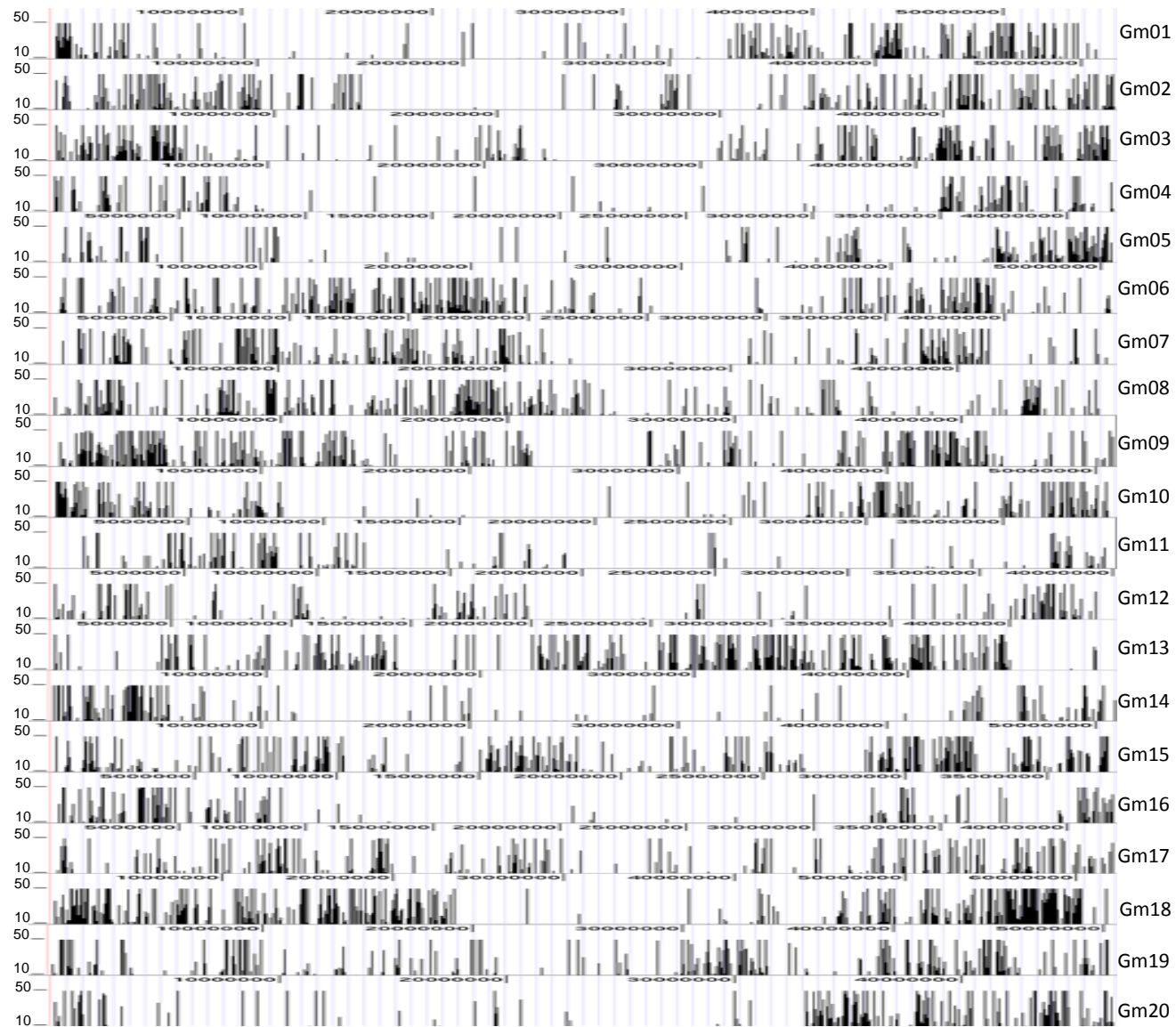

Supplement: Additional file 3 — Distribution of 7947 SNPs. This figure showed the distribution of the 7,947 SNPs predicted by strict stringency on the reference genome. The x-axis represents short read coverage, and the y-axis represents the SNP position on the chromosomes of the reference genome. [file 1471-2164-11-469-S3.PDF]
